# The complete mitochondrial genome of *Pedetontus zhejiangensis* (Microcoryphia: Machilidae) and its phylogeny

**DOI:** 10.1080/23802359.2020.1804472

**Published:** 2020-08-07

**Authors:** Shi-Qi Shen, Yin-Yin Cai, Ke-Ke Xu, Qing-Ping Chen, Si-Si Cao, Dan-Na Yu, Jia-Yong Zhang

**Affiliations:** a College of Chemistry and Life Science, Zhejiang Normal University, Jinhua, China; b Key Lab of Wildlife Biotechnology, Conservation and Utilization of Zhejiang Province, Zhejiang Normal University, Jinhua, China

**Keywords:** Microcoryphia, mitogenome, phylogeny, *Pedetontus zhejiangensis*

## Abstract

The complete mitochondrial genome of *Pedetontus zhejiangensis* (Microcoryphia: Machilidae) was successfully sequenced. The mitochondrial genome of *P. zhejiangensis* was a circular molecule of 15,602 bp in length, containing 13 protein-coding genes, 2 rRNA genes, 22 tRNA genes, and the control region, which showed the typical insect mitochondrial genome arrangement. The AT content of the whole genome was 73.8% and the length of the control region was 671 bp with 82.5% AT content. In BI and ML phylogenetic trees, *P. zhejiangensis* was a sister group to *Pedetontus silvestrii*, and the monophyly of *Pedetontus* was strongly supported. The genus *Pedetontinus* was a sister group to *Pedetontus*.

Microcoryphia is a primitive order in Insecta including two families Machilidae and Meinertellidae (Sturm and Machida 2001; Mendes 2002). To date, there are over 30 species described in China (Sturm and Machida 2001; Zhang et al. 2005; Zhang and Li 2009; Yu et al. 2010; Cheng et al. 2011; Deng et al. 2011; Zhang and Zhou 2011; Li et al. 2012). Whereas there are 10 mitochondrial genomes of Microcoryphia reported. In this study, we sequenced the mitochondrial genome of *Pedetontus zhejiangensis* to discuss the phylogenetic relationship within Microcoryphia.

The samples of *P. zhejiangensis* were collected from Mountain Laoshan (N32°6′25″, E118°36′28″), Nanjing Jiangsu Province, China. The samples were identified and stored at −40 °C in the Animal Specimen Museum, College of Life Sciences and Chemistry, Zhejiang Normal University, China. Total genomic DNA from one whole sample (LSTB20080707) was extracted using Ezup Column Animal Genomic DNA Purification Kit (Sangon Biotech Company, Shanghai, China) and stored in the Zhang laboratory. We amplified overlapping fragments of the *P. zhejiangensis* mitochondrial genome by normal PCR and LA-PCR methods according to Ma et al. (2015). Sequences were checked and assembled using SeqMan (Lasergene version 5.0) (Burland 2000). The genomic sequence has been deposited in GenBank with an accession number MT679724.

The complete mitochondrial genome of *P. zhejiangensis* was a typical circular DNA molecule of 15,602 bp in length containing 37 genes. The AT content of the whole genome was 73.8% and the length of the control region was 671 bp with 82.5% AT content. All 13 PCGs began with ATN (N represents A, T, G, and C) as the start codon. The *ATP6*, *ATP8*, *COII*, *ND2*, *ND3*, *ND4L*, *Cyt b*, *ND1*, and *ND6* genes were terminated with TAA as the stop codon, whereas the other PCGs (*COI*, *COIII*, *ND5*, and *ND4*) ended with the incomplete stop codon TA or T.

The mitochondrial genomes of 13 species were used to discuss the phylogenetic relationships, including 10 species of Microcoryphia as ingroups (Cameron et al. 2004; Podsiadlowski 2006; Carapelli et al. 2007; Zhang et al. 2008; He et al. 2013; Ma et al. 2015) and two outgroups (*Homarus americanus* and *Squilloides leptosquilla*; Kim et al. 2011). Bayesian inference (BI) and maximum likelihood (ML) trees were constructed using the 13 protein-coding genes of the nucleotide alignment. Each alignment was performed by Gblock 0.91b (Castresana 2000) using default settings to select conserved regions of the nucleotide. The first and second codon positions of protein-coding genes were used in the nucleotide dataset. BI and ML analysis was performed by MrBayes3.1.2 (Huelsenbeck and Ronquist 2001) and RAxML 8.2.0 (Stamatakis 2014), respectively. In BI and ML phylogenetic trees, *P. zhejiangensis* was a sister group to *Pedetontus silvestrii*, and the monophyly of *Pedetontus* was strongly supported (Figure 1). The genus *Pedetontinus* was a sister group to *Pedetontus*. The monophyly of Machilinae and Petrobiinae was not well supported as well as the results of Ma et al. (2015).

**Figure 1.**
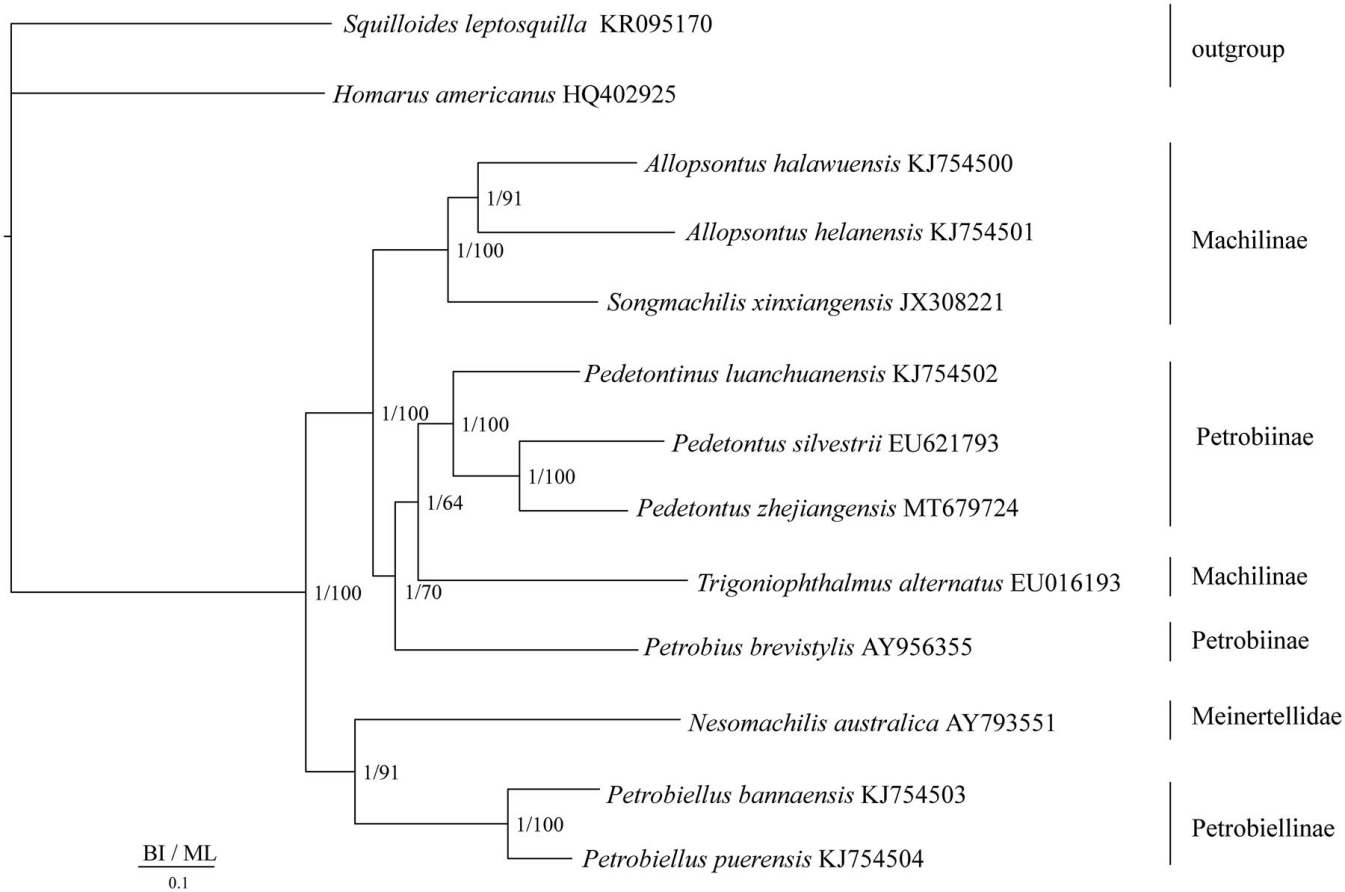
Phylogenetic tree of the relationships among 13 species of Microcoryphia, including *P. zhejiangensis* based on the nucleotide dataset of the 13 mitochondrial protein-coding genes. The Bayesian posterior probability values and the maximum-likelihood bootstrap values are indicated above nodes. The GenBank numbers of all species are shown in the figure.

## Data Availability

The data that support the findings of this study are openly available in NCBI at www.ncbi.nlm.nih.gov reference number [MT679724].The file was used to construct the phylogenetic relationship of bristletails at https://cloud.zjnu.edu.cn/share/e90464aaef1f9a9c2a2eb4fc9c
